# Retrograde pedal access and percutaneous mechanical thrombectomy for salvage of an occluded vein bypass

**DOI:** 10.1016/j.jvscit.2024.101510

**Published:** 2024-04-14

**Authors:** Jayne R. Rice, Louis Darkwa, Grace J. Wang

**Affiliations:** a Division of Vascular Surgery and Endovascular Therapy, Hospital of the University of Pennsylvania, Philadelphia, PA; b University of Illinois College of Medicine at Chicago, Chicago, IL

**Keywords:** Pedal access, Percutaneous mechanical thrombectomy, Retrograde access, Revascularization, Vein graft occlusion

## Abstract

Managing occlusions in a lower extremity bypass is challenging, although several surgical methods and percutaneous devices are available for treatment. A 64-year-old man presented with subacute failure of his infrainguinal vein bypass. Because we were unable to access the bypass in an antegrade fashion, we accessed the bypass graft via retrograde pedal access. The occluded vein graft was salvaged with the Pounce percutaneous mechanical thrombectomy system (Surmodics) with the use of a 0.014-in. through and through buddy wire to maintain access in the bypass alongside the Pounce system to allow multiple passes of the nitinol baskets to retrieve thrombus.

Managing occlusions in a lower extremity bypass is challenging. The ideal treatment aims for low reoperation rates with effective limb salvage and patency.^1^ In this case, we salvaged a bypass in a single operative room session with the use of a percutaneous mechanical thrombectomy system to retrieve subacute and chronic clot. The patient provided written informed consent for the report of his case details and imaging studies.

## Case report

A 64-year-old man with a history of diabetes and hypertension presented with a nonhealing transmetatarsal amputation (TMA) and 3 days of worsening rest pain. Two months prior, he underwent a left superficial femoral artery (SFA) to posterior tibial (PT) bypass with an ipsilateral reversed great saphenous vein, followed by TMA for tissue loss (WIfI [wound, ischemia, foot infection] score, 232). Completion angiogram performed after the original surgery demonstrated no anastomotic issues and good flow throughout the conduit with no areas of twist. The 1-month routine follow-up duplex ultrasound showed mildly elevated velocities in the low 200s in the inflow and outflow artery with, however, no other evidence of significant stenosis throughout the bypass. He reported being compliant with his dual antiplatelet therapy and continued to abstain from smoking. Computed tomography angiography was performed confirming the left SFA to PT bypass was occluded. He was heparinized and brought to the operating room the following day for arteriography and possible revascularization.

**Fig 1:**
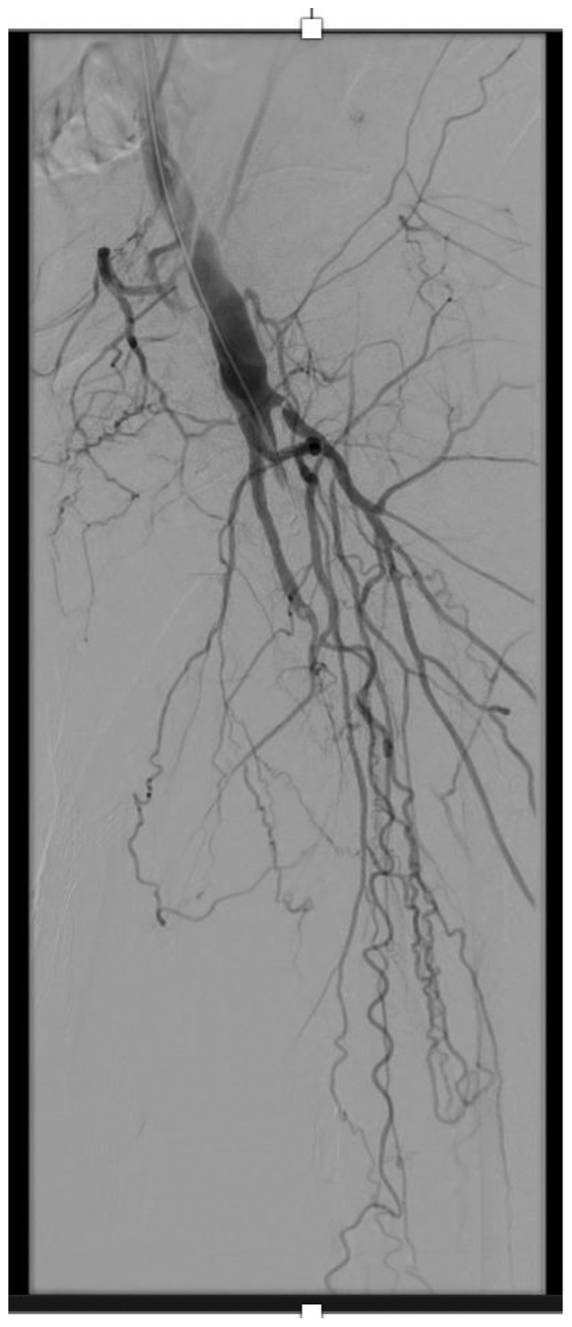
Antegrade angiogram of left common femoral artery. The profunda artery and proximal superficial femoral artery (SFA) are patent. The bypass is occluded, and the hood of the bypass cannot be visualized for access in the antegrade fashion.

The patient was induced under general endotracheal anesthesia. Ultrasound-guided access of the right femoral artery was obtained. Following access and systemic heparinization, a 7F sheath was inserted using the Seldinger technique over a Rosen wire. The common femoral artery and profunda artery were patent; however, the bypass was occluded, and the hood of the bypass could not be seen on angiography (Fig 1). With access in the left common femoral artery, attempts were made to access the proximal bypass; however, the native SFA and profunda artery were cannulated.

**Fig 2:**
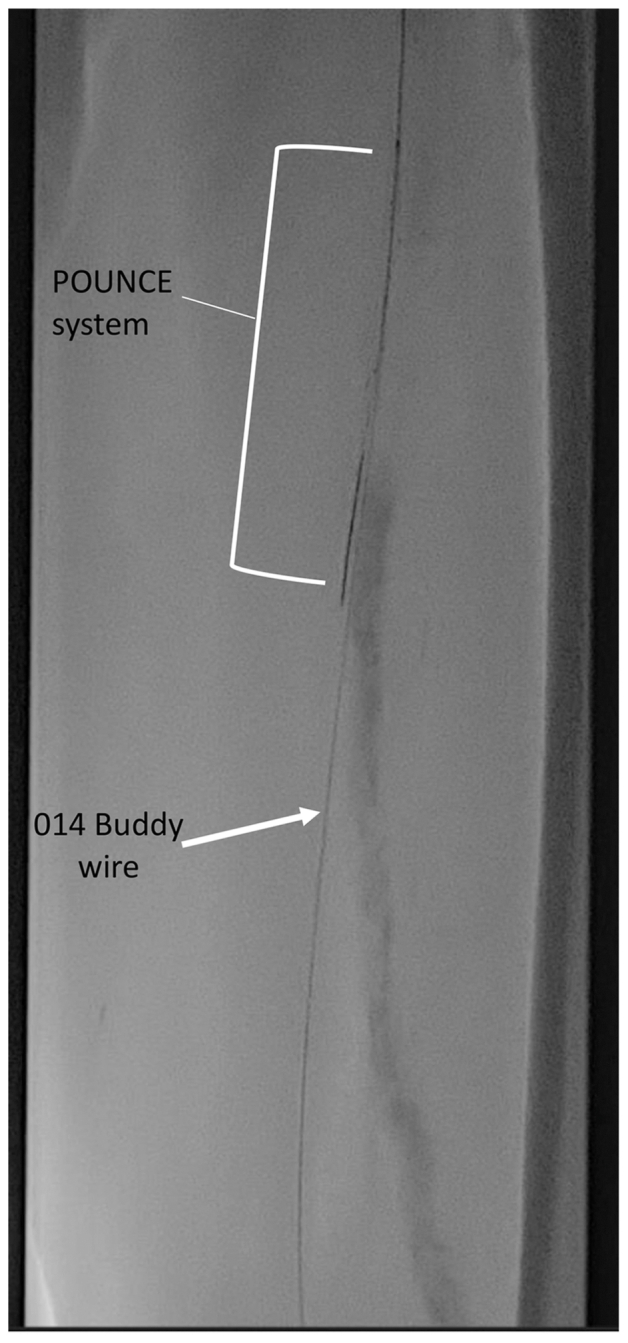
Pounce 0.018-in. wire and 0.014-in. buddy wire in the bypass.

**Fig 3:**
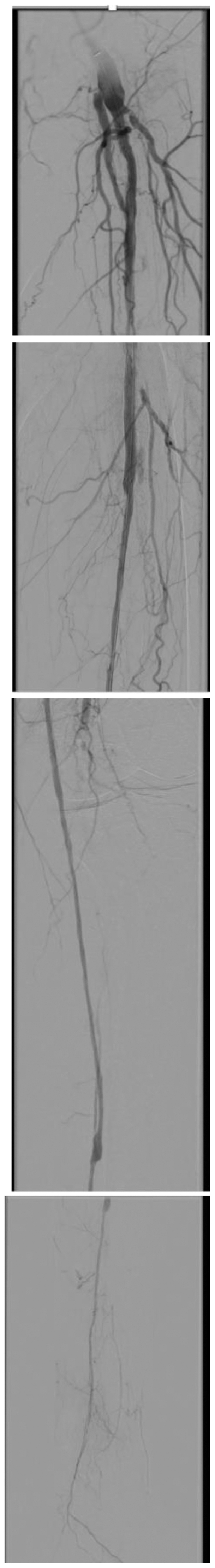
Final completion angiogram showing a patent bypass.

Because we could not access the bypass graft antegrade, we decided to access the bypass retrograde via the PT artery. The ankle was subsequently prepared, and the PT artery was accessed by the ankle using the pedal access kit. A 4F micropuncture sheath was then inserted. A Whisper wire (Abbott Cardiovascular) was readily advanced through the PT artery, through the vein bypass graft, and into the native SFA and common femoral artery. The wire was snared up and over the aortic bifurcation with a Amplatz GooseNeck snare catheter (Medtronic) from the 7F Destination sheath (Terumo Interventional Systems) to create a through and through wire from the right groin to the left ankle. With this through wire in place, a Penumbra CAT 6 catheter (Penumbra) was advanced via the contralateral access, and aspiration thrombectomy used to remove some acute clot. Arteriography at this time demonstrated that the bypass was now visible; however, residual clot was still present. We used the Pounce system (Surmodics) to remove the remaining acute and subacute clot in the bypass. The Pounce system was introduced over an 0.018-in. wire alongside the buddy 0.014-in. through and through wire (Fig 2). This allowed us to maintain wire access in the bypass via multiple passes with nitinol baskets. After multiple thrombectomy passes, angiography demonstrated excellent results. However, residual clot and occlusive disease were still present in the proximal and distal anastomosis of the bypass. Balloon angioplasty of the proximal anastomosis was then performed using a 6 × 60 Ranger balloon (Boston Scientific), along with placement of a 6 × 100 Viabahn stent (W.L. Gore & Associates) from the native SFA into the proximal graft. The distal anastomosis was likewise ballooned using a 2.5-mm Coyote balloon (Boston Scientific). A completion angiogram showed a patent bypass with excellent angiographic results (Fig 3). Hemostasis was obtained in the PT artery with prolonged angioplasty, and the right groin was percutaneously closed with a Perclose Proglide device (Abbott Cardiovascular). The patient was given 500 U/hour of heparin overnight and a full dose of heparin on postoperative day 1. He underwent redo TMA on postoperative day 4. The patient progressed well postoperatively and was discharged with a prescription for clopidogrel (Plavix; Bristol-Myers Squib – Sanofi Pharmaceuticals) and apixaban (Eliquis; Bristol-Myers Squibb). At the patient's 3-month postoperative visit, the bypass was still patent, and his TMA has healed.

## Discussion

In the subacute period, failure of a bypass can be due to anatomical lesions such as stenosis of inflow or outflow or technical problems such as an unrecognized sclerotic segment of vein or frozen valve.^2^ The failure of this bypass was most likely due to severe outflow disease, as seen on the final angiogram (Fig 3). Options for managing an occluded bypass include catheter directed thrombolysis therapy (CDTT), percutaneous mechanical and aspiration thrombectomy, and, when necessary, surgical revision or replacement of the graft. CDTT is often used as first-line treatment of acute infrainguinal bypass occlusion and has been shown to result in successful reopening of occluded bypasses and subsequent resolution of ischemic symptoms.^3^^,^^4^ However, this technique is fraught with complications in the short and long term, including premature cessation of thrombolysis and bleeding complications. CDTT is also associated with additional costs due to multiple trips to the operating room and because some institutions require intensive care unit stay during lysis. Finally, some patients have a contraindication to lysis, given their comorbidities or challenges with compliance in lying flat during the lysis process.^5^ The present patient specifically could not tolerate lying flat for >2 hours.

Therefore, mechanical thrombectomy and aspiration thrombectomy provide an additional endovascular recanalization technique that has been shown to have better technical success and limb salvage rates than thrombolysis.^6^^,^^7^ In a prospective study, Gray et al^8^ reports success removing thrombus from the SFA and popliteal artery segment and a reduction in the need for concomitant CDTT in 44 of 46 patients. Unlike other percutaneous thrombectomy methods such as Penumbra suction thrombectomy (Penumbra Inc), Pounce offers a distinct advantage by effectively addressing subacute and chronic clots, expanding its utility in a broader range of clinical scenarios.^8^ However, it is worth noting that the Pounce system does present with a unique challenge—potential wire access loss within the targeted lesion and the need to rewire the artery after each retrieval. To address this issue, we implemented a buddy wire strategy placed in a retrograde fashion. Alongside the 0.018-in. wire system necessary for deploying the baskets and retrieving the thrombus, a 0.014-in. buddy wire was retained. This dual-wire approach ensures continued access to the area of the lesion, allowing multiple passes to be performed to effectively fully evacuate subacute and chronic thrombi, which are often more challenging to evacuate. Following mechanical thrombectomy with the percutaneous thrombectomy system, adjuvant angioplasty and stenting was performed to treat residual lesions and stenosis in the proximal and distal anastomoses.

The findings from this study add to those of previous studies that have reported successful revascularization with the percutaneous thrombectomy system. Current reports at the time of writing have only used the percutaneous thrombectomy system in native vessels. We were able to successfully use the Pounce system in the salvage of a vein graft without the need for additional thrombolysis. Although the cost of using multiple devices and implants should be considered in the decision to proceed in this fashion, rather than with thrombolysis, this single-session limb salvage procedure effectively addressed our patient’s acute on chronic limb-threatening ischemia issues, without prolonging his length of stay.

## Conclusions

We have demonstrated technical success in the salvage of an infrainguinal vein bypass via contralateral groin antegrade access and retrograde pedal access with the Pounce system and a 0.014-in. buddy wire to maintain wire access in the bypass for multiple thrombectomy passes.

## Disclosures

None.
